# Comparison of Antioxidant Capability after Isopropanol Salting-Out Pretreatment and *n*-Butanol Partition Extraction, and Identification and Evaluation of Antioxidants of *Sedum formosanum* N.E.Br.

**DOI:** 10.3390/molecules21040513

**Published:** 2016-04-19

**Authors:** Jung-Hui Chen, Wen-Hui Lai, Shang-Dung Lin, Cheng-Fong Lan, Shih-Lan Hsu, Ming-Yuan Liao

**Affiliations:** 1 Department of Mechanical Engineering, Nan-Kai University of Technology, 568 ZhongZheng Road, Caotun, Nantou County 542, Taiwan; 2 Department of Chemistry, National Chung Hsing University 250 Kuo-Kuang Road, Taichung 402, Taiwan; David751008@gmail.com (W.-H.L.); gloom2007@hotmail.com (S.-D.L.); mark921117@gmail.com (C.-F.L.); 3 Department of Medical Research, Taichung Veterans General Hospital, 1650 Taiwan Road Sec, Taichung 402, Taiwan; h2326@vghtc.gov.tw

**Keywords:** *S. formosanum* N.E.Br., salting-out, cyanophoric glycoside, flavonoids, antioxidant activity

## Abstract

Crude extracts of *Sedum formosanum* N.E.Br. obtained from *n*-butanol partition (BP) and isopropanol salting-out pretreatment (ISP) were analyzed using antioxidation assays. The results indicated that the extract from ISP contained more potent antioxidants and thus exhibited more antioxidant activity in all the assays. The superoxide radical-scavenging activity and inhibition of nitric oxide radicals achieved after ISP were 3.65 and 2.18 times higher than those achieved through BP, respectively. Eight bioactive natural products were isolated and identified according to an analysis of antioxidation activity in different fractions of the ISP crude extract, namely three cyanophoric glycosides **1**–**3**, three flavonoids **4**–**6** and two phenolic compounds (**7** and a new compound **8**). Among them, compounds **5** and **6** exhibit the highest antioxidation capability, and the ISP is suitable for obtaining compounds **5** and **6** using HPLC chromatograms. Therefore, ISP is an excellent extraction technology that can be used to extract antioxidant compounds in the nutraceutical and pharmaceutical industries.

## 1. Introduction

In the human body, the normal oxidative metabolism constantly produces reactive oxygen species (ROSs), such as hydrogen peroxide, superoxide (O_2_**^.−^**), the hydrogen radical (**^.^**OH), singlet oxygen, and nitrogen species. *In vivo*, through an endogenous antioxidant defense mechanism comprising antioxidant enzymes, such as superoxide dismutase, catalase, and glutathione peroxidase, are firmly coupled at their generation site or are detoxified, possibly preserving optimal cellular function [1]. However, under pathological conditions, detoxifying mechanisms are often inadequate, possibly producing excessive quantities of ROS. Oxidative stress is a pro-oxidant shifting process that can degrade cellular components, such as DNA, carbohydrates, polyunsaturated lipids, and proteins. Furthermore, upsetting the pro-oxidant-antioxidant balance generates oxidative stress. This oxidative stress can cause enzyme inactivation, irreversible cellular dysfunction, and cell death [2]. Antioxidant compounds can inhibit the initiation or propagation of oxidation chain reactions and delay or inhibit the oxidation of lipids or other molecules. Therefore, antioxidant compounds have received increasing attention regarding their ability to prevent or repair oxidative damage [3]. Because chemically produced analogs are reported carcinogens, natural antioxidants are likely to be acceptable to users [4].

The phytochemicals of plants are potential sources of natural antioxidants, including phenolic compounds, flavonoids, alkaloids, and terpenoids [5]. In particular, flavonoids and phenolic compounds are highly effective antioxidants that possess anticancer, hypolipidemic, anti-aging, and anti-inflammatory properties; thus, they have received increasing attention [6]. Moreover, flavonoids can protect biological systems by scavenging free radicals, chelating metal catalysts, activating antioxidant enzymes, reducing α-tocopherol radicals, and inhibiting oxidase capability [7]. Flavonoids are biodegradable and non-toxic, and they may be an appealing alternative to currently available commercial synthetic antioxidants [8].

The genus *Sedum* (Crassulaceae), a medicinal plant, grows mainly in various Eastern-European regions and on mountain slopes in China and South Korea, and numerous *Sedum* species are used pharmaceutically. *Sedum kamtschoticum* Fischer is a perennial common in South Korea, China, and Japan. Its water extracts have been used in folk medicine, particularly as anti-anxiety, anti-inflammation, and analgesic agents and for improving blood circulation [9]. *Sedum dendroideum* Moc & Sessé is widely used to treat ulcers, inflammation, and wounds in Brazil and other parts of the world [10]. *Sedum telephium* possesses local anti-inflammatory activity and is presently used throughout Europe for healing wounds and treating various types of local inflammation [11]. *Rhodiola quadrifida* (PAll) Fish. et Mey is prescribed for hemostatic, antibechic, and tonic uses in Chinese medicine, is used for preparing endermic liniments for burns and contusions, and was shown to possess antiallergic activity in rats in a passive cutaneous anaphylaxis test [12]. *Sedum sarmentosum* is a type of folk medicine that has been used to treat chronic viral hepatitis in China and South Korea. The plant granules have been used in the clinic since 1971 [13], and inhibit oleic-acid-albumin-induced lipid accumulation in HepG2 cells [14]. Furthermore, this herb improves the survival of hepatoma patients by inhibiting excessive tumor cell growth [15]. In previous phytochemical studies on the *Sedum* species, several compounds have been isolated, such as alkaloids, tannins, flavonoids, megastigmene, and cyanogenic compounds [16,17,18]. *S. formosanum* N.E.Br. is a perennial herbaceous plant that grows mainly in wetlands, coastal areas, or seams of rock and gravel. Although *S. formosanum* is widely distributed in Asia, the components responsible for the antioxidant activity of *S. formosanum* have not been explored in detail. Therefore, further research must be performed to isolate and identify these plant extract components for nutraceutical and pharmaceutical applications.

This study was performed in three parts. First, we evaluated the antioxidant activity of the crude extracts of *S. formosanum* N.E.Br. obtained by using isopropanol salting-out pretreatment (ISP) and *n*-butanol partition (BP) extraction technology. Next, we isolated and identified the major compounds from the previous crude extracts and evaluated their antioxidant activity by using different antioxidant assays. In the final stage, we compared the chromatograms of the crude extracts obtained with the ISP and BP technology, and demonstrated that the hydrophilic ISP extraction technology is superior to the BP extraction technology for promoting antioxidant extraction. The antioxidant-promotion capability, such as the chelation of ferrous as well as nitric oxide (NO) and superoxide anion radical scavenging activity, were compared between the ISP and SP extraction technology for the first time. The purpose of this study was to confirm the value of the ISP extraction technology and to prove that this technology can be universally applied for extracting and isolating natural plants for nutraceutical and pharmaceutical applications.

## 2. Results and Discussion

### 2.1. Comparison of the Antioxidant Capability of n-Butanol Partition and Isopropanol Salting-Out Pretreatment Extraction Technology

Table 1 displays the antioxidant capability results after use of the ISP and BP extraction technology, which were obtained using assays on superoxide radical-scavenging activity, oxygen radical absorbance capacity (ORAC) radical-scavenging activity, chelation of ferrous ions, and inhibition of NO radical activity. The antioxidant activity of the crude extract is expressed in micromoles of Trolox equivalent (TE) per gram of dried materials. After statistical calculation, the *t*-test values are 2.442, 43.268, 15.08 and 22.624 for FRAP, SOD, NO and ORAC, respectively. Therefore, the antioxidant capability comparison of the ISP and BP extractions possesses statistical significance. The results indicated that the antioxidant capability of the crude extract from the ISP extraction technology was between 3.65 and 1.38 times higher than that of the crude extract obtained by the BP extraction technology. The superoxide radical-scavenging activity and inhibition of the NO radical obtained using ISP extraction technology were respectively 3.65 and 2.18 times higher than those obtained using BP extraction technology (0.21 *vs.* 0.057 micromoles of TE/g; 0.118 *vs.* 0.054 micromoles of TE/g, respectively). These data suggest that ISP extraction technology is preferable to BP extraction technology for extracting antioxidants. Consequently, isolating and evaluating the antioxidant capability of the pure compounds of *S. formosanum* N.E.Br. by using ISP extraction technology is significant.

### 2.2. Identification of Compounds ***1**–**8*** Using Isopropanol Salting-Out Pretreatment Extraction Technology

**Figure 1:**
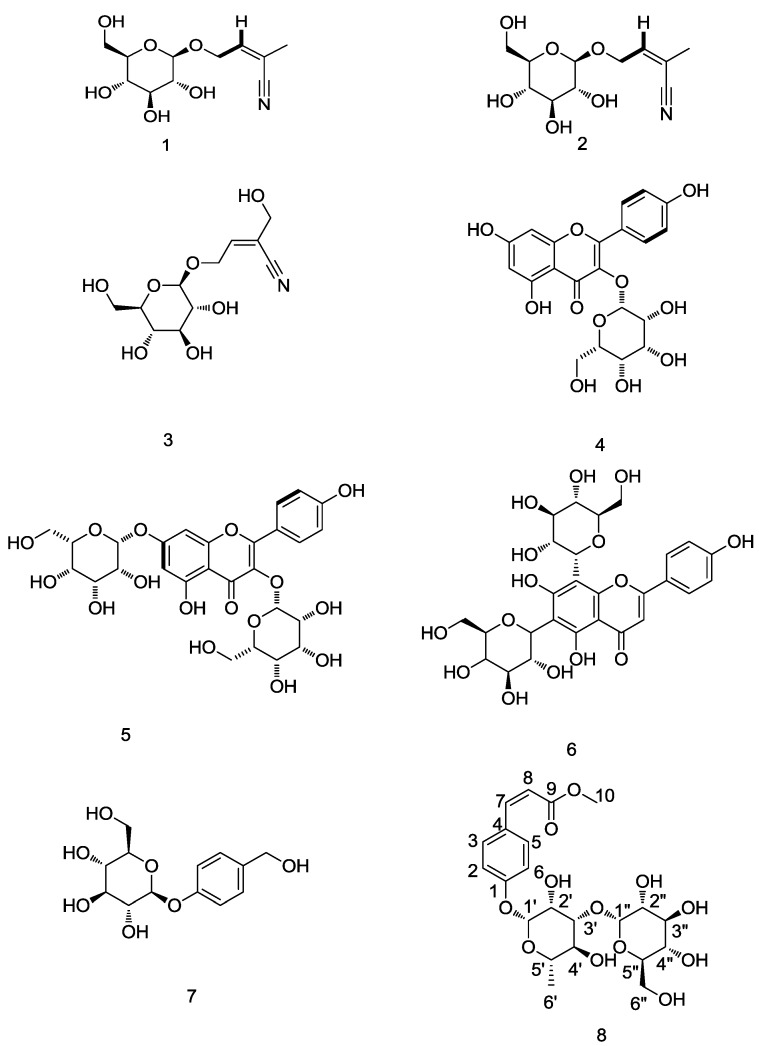
Chemical structures of compounds **1**, **2**, **3**, **4**, **5**, **6**, **7** and **8**.

Three cyanophoric glycosides, three flavonoids, and two phenolic compounds were isolated from *S. formosanum* N.E.Br by using isopropanol salting-out extraction technology. Their structures were determined as rhodiocyanoside A (**1**), rhodiocyanoside D (**2**), sarmentosin (**3**), kaempferol-3-*O*-β-d-glucopyranoside (**4**), kaempferol-3,7-di-*O*-β-d-glucopyranoside (**5**), vicenin-2 (**6**), gastrodin (**7**), and cis-*p*-coumaric acid-4-β-*O*-l-glucopyranosyl-(1→3)-α-l-rhamnopyranoside methyl ester (**8**). Compound **8** is a new compound, whereas compounds **1**, **2**, **3**, **5**, **6** and **7** were identified for the first time from *S. formosanum* N.E.Br. Their structures are shown in Figure 1.

**Figure 2:**
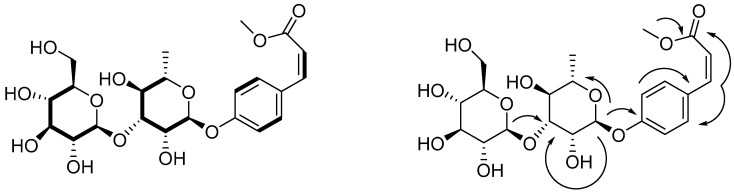
^1^H-^1^H COSY and HMBC correlation of compound **8**.

The molecular formula of compound **8** is C_22_H_30_O_12_ deduced from HR-FT-MS ([M + H]^+^ *m*/*z*: 487.1812). The IR spectrum of compound **8** revealed the presence of the hydroxyl group (3362 cm^−1^) and α,β*-*unsaturated ester C=O group (1660 cm^−1^). The ^1^H-MNR and ^1^H-COSY spectra (Table 2 and Figure 2) suggest the presence of a *para*-substituted phenyl ring, A_2_B_2_ protons at δ 7.05 (d, *J* = 8.7 Hz, H_2,6_) and δ 7.66 (d, *J* = 8.7 Hz, H_3,5_), two single protons coupled to double doublets δ 6.92 (d, *J* = 12.5 Hz, H-7) and δ 5.87 (d, *J* =12.5 Hz, H-8), in which *J* = 12.5 Hz is the cis-form. One methoxyl group at δ 3.70 (3H, s) and in conjunction with the ^13^C-NMR signals at 158.4 (C_1_), 116.8 (C_2,6_), 133 (C_3,5_), 129.4 (C_4_), 144.2 (C_7_), 118.2 (C_8_), 165.8 (C_9_) and 51.8 (C_10_) indicated that compound **8** is cis-*p*-coumaric acid methyl ester. The ^1^H-NMR and ^13^C-NMR spectra of compound 8 revealed the presence of a α-l-rhamnopyranosyl moiety [δ_H_ 5.51 (d, *J* = 1.8 Hz, H-1’) and δc 99.3, 71.2, 82.5, 72.5, and 18.1] and a β-d-glucopyranosyl moiety [δ 4.60 (d, *J* = 7.5 Hz, H-1’’) and δc 105.9, 72.3, 77.7, 72.6, 77.8, and 62.0]. In HMBC experiment, between the H-1” of the d-glucose and the C-3’ of the l-rhamnose, between the H-1’ of the l-rhamnose and the C-1 of compound 8, and between the H of methoxyl and the C-9 of compound 8 had long-range correlation (Figure 2) and assignment confirmed by decoupling, ^1^H-^1^H COSY, HMQC and HMBC. Hence, compound **8** was elucidated to be cis-p-coumaric acid-4-β-*O*-l-glucopyranosyl-(1→3)-α-l-rhamnopyranoside methyl ester.

### 2.3. Antioxidant Activity Potentials of Identified Compounds 

Table 3 shows the superoxide radical-scavenging activity, ORAC radical-scavenging activity, chelation of ferrous ions, and inhibition of NO activity of compounds **1**–**8** that were determined using ISP extraction technology for *S. formosanum* N.E.Br.

Compounds **1**–**3** are cyanogenic glycosides, Compounds **4**–**6** are flavonoid glycosides, and compounds **7** and **8** are other compounds. Compounds **4** and **5** are kaemferol glycosides, whereas compound **6** is a flavone glycoside. The radical-scavenging activity of these compounds was ranked as **7** > **6** > **5** > **4** > **2** > **8** > **1** > **3** in the superoxide radical-scavenging activity assay; **6** > **5** > **4** > **8** in the ORAC radical-scavenging activity assay; **6** > **5** > **4** > **7** > **8** > **2** > **3** > **1** in the assay on the chelation of ferrous ions; and **5** > **6** > **4** > **8** > **7** > **1** > **2** ≈ **3** in the NO activity inhibition assay. Among these compounds, compounds **6** and **5** exhibited the highest antioxidant capability, as determined using the afore-mentioned methods (superoxide radical-scavenging activity: 1.03 *vs.* 0.95 mmol of TE; ORAC radical-scavenging activity: 1.06 *vs.* 1.03 mmol of TE; chelation of ferrous ions: 1.23 *vs.* 1.14 mmol of TE; and inhibition of NO activity: 0.83 *vs.* 1.01 mmole of TE, respectively). As mentioned previously, the structural characteristics of the analytes are mainly crucial to determining antioxidant capability.

Compounds **4**–**6** contain similar aglycon structures (the C-ring 2,3-double bond does not link the OH in position 3 of compound **6**), but at different positions and exhibit the conjugation of one or two sugar moieties. Thus, it is presumed that the solubility of these compounds [19], steric effects, and the degree of facilitation of the delocalization of electrons from the B-ring to the C-ring may determine their antioxidant capability [20]. Compounds **4** and **5** have identical structures (one sugar linked at the C3 position); however, compound **5** has one more sugar link at the A7 position. Compound **5** has improved solubility in an aqueous solution, facilitating the delocalization of the electrons from the B-ring to the C-ring. Consequently, compound **5** has higher antioxidant capability than compound **4** does. Although compounds **5** and **6** have equal numbers of sugars (linked at different positions of the aglycon structure), in compound **5**, the one sugar link at the C3 position of the C-ring at the 2,3-double bond causes steric hindrance and may interrupt the delocalization of the electrons from the B-ring to the C-ring. Hence, compound **6** was more efficient than compound **5** in the antioxidant capability assays, except for NO inhibiting activity. Compound **5** is traditionally used for treating kidney diseases among Mexican natives and has been isolated and identified from certain ferns [21,22].

The linking of cyanide at different positions of the double bond may cause differences in antioxidant capability among compounds **1**–**3**. Cyanogenic glycosides has little toxicity and are crucial compounds for pharmaceutical use. Compound **1** is the main natural medicine constituent of *R. quadrifida* (Pall.) Fisch. et Mey which inhibits histamine release with anti-2,4-dinitrophenyl IgE from sensitized rat peritoneal exudate cells, and exhibits antiallergic activity in rats, according to a passive cutaneous anaphylaxis test [23]. Compound **3** is the main active constituent of *S. sarmentosum* and is used to treat chronic viral hepatitis in Asia [15].

Compound **8** is a *p*-coumaric acid derivative, and compound **7** is a *p*-hydroxybenzyl alcohol derivative. The antioxidant capability of compound **7** is higher than that of compound **8**, except for inhibition of NO activity. Compound **7** is the main active constituent of *Rhizoma gastrodiae*, and is considered a traditional Chinese medicine proven to be an effective and safe drug for clinical use to prevent neurocognitive decline following cardiopulmonary bypass, and benefitting older refractory hypertension patients [24]. This compound can improve the association between endothelin and NO in plasma [25].

### 2.4. Comparison of the Chromatograms after Isopropanol Salting-Out Pretreatment and n-Butanol Partition Extraction Technology Using HPLC Separation

**Figure 3:**
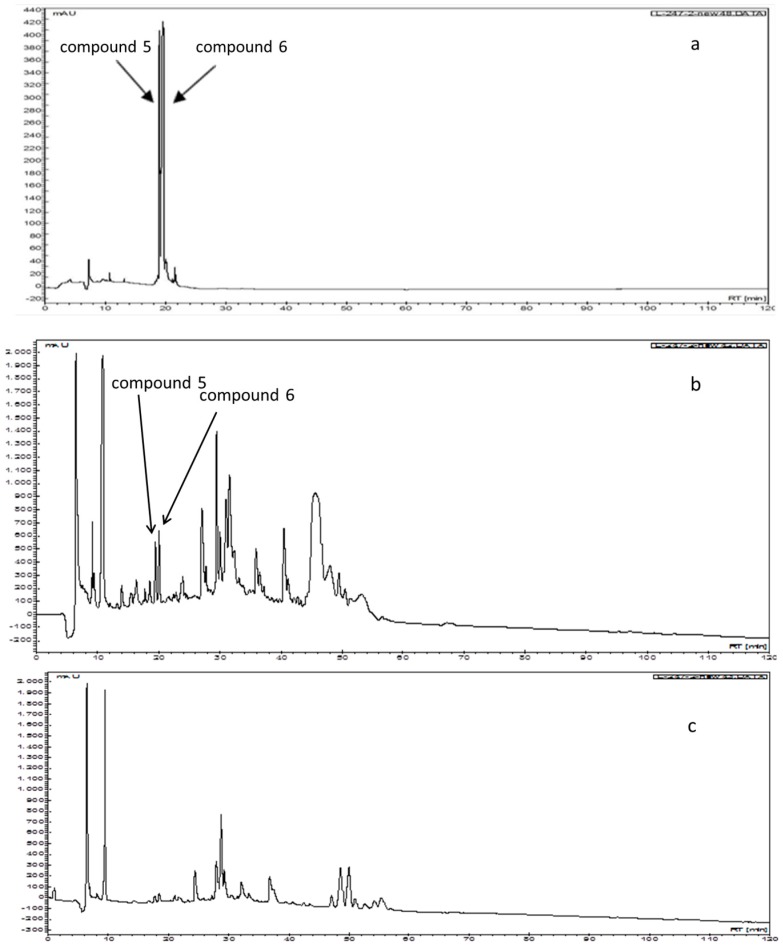
Analytical HPLC chromatogram monitored using λ_210_ UV absorption: (**a**) compounds **5** and **6**; (**b**) isopropanol salting-out extracts of *S. formosanum* N.E.Br; (**c**) *n*-butanol partition extracts of *Sedum formosanum* N.E.Br.

Figure 3a reveals that the retention times (t_R_, min) of compounds **5** and **6**, which exhibited the highest antioxidant activity in all of the antioxidant assays, were 19.0 and 20.0 min, respectively. The HPLC chromatograms of the separation of the compounds that were obtained using the ISP and BP extraction technology differ widely (Figure 3b,c). The ISP extraction technology is suitable for obtaining compounds **5** and **6**. This indicates that the antioxidant capability, achieved using ISP extraction technology was 1.38–3.65 times higher than that achieved using BP extraction technology. 

## 3. Materials and Methods

### 3.1. General Procedures

Through the extraction and isolation of solvents, analytical reagents of various grades, including isopropanol, dichloromethane, methanol, and *n*-butanol, were obtained from ECHO (Miaoli, Taiwan). HPLC grade acetonitrile (ACN) was purchased from Merck Chemicals (Darmstadt, Germany). KH_2_PO_4_, K_2_HPO_4_, phosphate-buffered saline, and the NMR solvents, methanol-*d*_4_, DMSO-*d*_6_, nitroblue tetrazolium (NBT), nicotinamide adenine dinucleotide (NADH), phenazine methosulfate (PMS), ferrous chloride, ferrozine, sodium nitroprusside (SNP), 2,2-azobis(2-amidopropane) dihydrochloride (AAPH), sulfanilamide, and napthyethylenediamine dihydrochloride (NED), were obtained from Sigma-Aldrich Chemical Co. (St. Louis, MO, USA). Trolox and fluorescein disodium were obtained from Aldrich (Milwaukee, WI, USA). Ultra-pure water (>18 MΩ) was obtained using a SG-Ultra water purification system (SG Water USA, LLC, USA), degassed under vacuum, and filtered through a 0.45 μm membrane filter before use. Sodium chloride was purchased from Union Chemical Works Ltd. (Hsinchu, Taiwan).

IR spectra were obtained using a Spectrum 100 FT-IR spectrometer (Perkin-Elmer, Wellesley, MA, USA). UV spectra were obtained using a U-300 spectrophotometer (Perkin-Elmer) with spectroscopy-grade methanol (Merck). ^1^H-NMR and ^13^C-NMR spectra were measured using a Innova 400 spectrometer (Varian, Palo Alto, CA, USA). The chemical shift values of the ^1^H- and ^13^C-NMR spectra are presented as δ (ppm) with TMS as the internal standard. ESI-MS data were recorded on a LCQ instrument (Thermo-Finnigan, San Jose, CA, USA), and HR-FT-MS data were measured using a JMS-SX/SX 102A tandem mass spectrometer (JEOL Ltd., Tokyo, Japan). HPLC data were obtained using the Varian ProStar 240 Solvent Delivery Module.

Silica gel 60 (Merck 70–230 mesh, 230–400 mesh, ASTM) and Sephadex-LH-20 (Pharmacia, Uppsala, Sweden) were used in column chromatography. The radical-scavenging activity assays were performed using an ELISA reader. 

### 3.2. Sources of Sedum formosanum

Taichung Veterans General Hospital in Taichung, Taiwan, provided the *S. formosanum* N.E.Br. samples. These samples were dried in an oven at 40 °C and stored overnight before extraction. 

### 3.3. Determination of the Two Types of Extraction Technology

#### 3.3.1. Isopropanol Salting-Out Pretreatment Extraction Technology 

The *S. formosanum* N.E.Br. powder (1 g) in aqueous 60% methanol (10 mL) was sonicated for 1 h at room temperature. Filter paper (Toyo Roshi Kaisha, Ltd., Tokyo, Japan) was used to filter the solution, which was then poured into a 50-mL round-bottomed flask. The residues were extracted twice by using 10 mL of aqueous 60% methanol (2 × 10 mL). Under reduced pressure, the extract was evaporated, the methanol was removed, and water was added to yield a 50 mL aqueous solution. Subsequently, isopropanol (50 mL) and NaCl (12 g) were added to this aqueous solution, and this solution was separated to yield the isopropanol fraction. Under reduced pressure and at a temperature of 40 °C, this fraction was evaporated to yield a dry residue [26,27]. Using water, this residue was dissolved to prepare a working solution (1000 mg/mL) for determining the antioxidant capability. 

#### 3.3.2. *n*-Butanol Partition Extraction Technology

The *S. formosanum* N.E.Br. powder (1 g) in aqueous 60% methanol (10 mL) was sonicated for 1 h at room temperature. Using filter paper (Toyo Roshi Kaisha, Ltd.), the solution was filtered and then poured into a 50-mL round-bottomed flask. The residues were extracted twice by using aqueous 60% methanol (2 × 10 mL). Under reduced pressure, the extract was evaporated, the methanol was removed, and water was added to yield a 50 mL aqueous solution. Next, *n*-butanol (50 mL) was added to this aqueous solution to yield the *n*-butanol fraction. Under reduced pressure and at a temperature of 40 °C, the *n*-butanol fraction was evaporated to yield a dry residue. This residue was dissolved in water, and a working solution (1000 mg/mL) was prepared to determine the antioxidant capability.

### 3.4. Extraction and Purification

The *S. formosanum* N.E.Br. powder (238 g) was extracted four-times at room temperature for 24 h using aqueous 60% methanol (400 mL). The extract was filtered, the methanol was removed through reduced pressure evaporation, and water was added to obtain a 1 L aqueous solution. An aliquot (200 mL) of the aqueous solution, isopropanol (200 mL) and sodium chloride (40 g) were added, and the solution was then separated to obtain isopropanol layers and aqueous layers. The isopropanol layers were collected, and a crude extract (33.6 g) was yielded through reduced pressure evaporation. The isopropanol extract (33.6 g) was chromatographed on silica gel and eluted using a gradient of CH_2_Cl_2_–CH_3_OH–H_2_O (from 89:10:1 to 59:40:1) to yield three fractions. The antioxidant capability of fractions 2 and 3 was higher than that of fraction 1, as determined using antioxidant assays (data not show).

Fraction 2 was chromatographed using a Sephadex-LH-20 column and eluted using a H_2_O–CH_3_OH gradient (from 100:0 to 0:100) to obtain two fractions. Fraction 1 was purified using semi-preparative HPLC with H_2_O and ACN as an eluted solvent at a flow rate of 2 mL/min to obtain rhodiocyanoside A (**1**, 3.6 mg), rhodiocyanoside D (**2**, 3.0 mg), sarmentosin (**3**, 10.0 mg). Fraction 2 was purified using semi-preparative HPLC with H_2_O and ACN as an eluted solvent at a flow rate of 2 mL/min to yield gastrodin (**7**, 1.0 mg), kaempferol-3-*O*-β-d-glucopyranoside (**4**, 3.4mg), and *cis-p*-coumaric acid-4-β-*O*-l-glucopyranosyl-(1→3)-α-l-rahmnopyranoside methyl ester (**8**, 2.7 mg).

**Figure 4:**
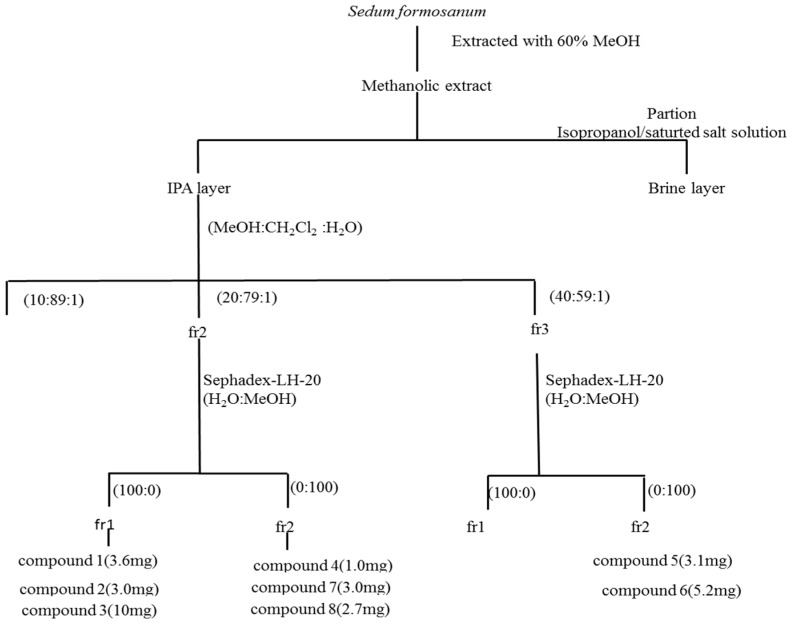
A flow chart the isolation and analytical sequences.

Fraction 3 was chromatographed using a Sephadex-LH-20 column and eluted using a H_2_O–CH_3_OH gradient (from 100:0 to 0:100) to obtain four fractions. Fraction 2 was purified using semi-preparative HPLC with H_2_O and ACN as an eluted solvent at a flow rate of 2 mL/min to obtain kaempferol-3,7-di-*O*-β-d-glucopyranoside (**5**, 3.1 mg) and vicenin-2 (**6**) (5.2 mg). Figure 4 is a flow chart that displays the isolation and analytical sequences. To examine the antioxidant capability of the compounds, methanol of each compound was dissolved to prepare a work solution (1000 mg/mL).

### 3.5. Compound Characterization

Compound **1**: C_11_H_16_NO_6_, white powder, UV $\lambda_{\max}^{\mathrm{MeOH}}$ nm: 208. ESI-MS: 282 [M + Na]^+^, IR ν_max_ cm^−1^: 3410, 2222, 1655, and 1076. ^1^H-NMR (methanol-*d*_4_): 6.46 (1H, qt, *J* = 1.2, 6.4 Hz, H-3), 4.42 (1H, ddq, *J* = 1.2, 6.4, 13.6 Hz, H-4a), 4.54 (1H, ddq, *J* = 1.2, 6.4, 13.6 Hz, H-4b), 1.98 (1H, *J* = 1.2 Hz, H-5), β-d-glucopyranosyl moiety: 4.29 (1H, *J* = 7.6 Hz, H-1’), 3.18~3.37 (m, glucose proton overlapped by water proton), 3.67 (1H, dd, *J* = 5.6, 12.6Hz, H-6’a), 3.86 (1H, dd, *J* =1.2, 12.6 Hz, H-6’b). ^13^C-NMR (methanol-*d*_4_): 118.1, 112.6, 147.0, 71.4, 71.4, 20.2, β-d-glucopyranosyl moiety: 104.0, 75.0, 78.0, 78.0, 71.4, 62.6, ppm. Moreover, the ^1^H-NMR and ^13^C-NMR data of compound **1** are consistent with existing literature [12]. Therefore, on the basis of these data, compound **1** was determined as rhodiocyanoside A.

Compound **2**: C_11_H_16_NO_6_, white powder, UV $\lambda_{\max}^{\mathrm{MeOH}}$ nm: 211. ESI-MS: 282 [M + Na]^+^, 725 [M − H]^−^, IR ν_max_ cm^−1^:3401, 2224, 1655, and 1076. ^1^H-NMR (methanol-*d*_4_): 6.71 (1H, q, *J* = 6.8 Hz, H-3), 4.24 (1H, ddq, *J* = 1.2, 6.4, 13.6 Hz, H-4a), 4.41 (1H, ddq, *J* = 1.2, 6.4, 13.6 Hz, H-4b), 2.01 (1H, d, *J* = 6.8 Hz, H-5), β-d-glucopyranosyl moiety: 4.29 (1H, d, *J* = 7.6 Hz, H-1’), 3.20~3.37 (m, glucose proton overlapped by water proton), 3.67 (1H, dd, *J* = 5.6, 12.0 Hz, H-6’a), 3.86 (1H, dd, *J* =1.6, 12.0 Hz, H-6’b). ^13^C-NMR (methanol-*d*_4_): 117.4, 114.0, 148.1, 71.4, 71.4, 20.2, β-d-glucopyranosyl moiety: 103.2, 74.9, 78.0, 78.0, 71.5, 62.7 ppm. Moreover, the ^1^H-NMR and ^13^C-NMR data of compound **2** are consistent with existing literature [23]. Therefore, on the basis of these data, compound **2** was determined as rhodiocyanoside D.

Compound **3**: C_11_H_17_NO_7_, colorless gum, UV $\lambda_{\max}^{\mathrm{MeOH}}$ nm: 212. ESI-MS: 298 [M + Na]^+^, IR ν_max_ cm^−1^: 3540~3240, 2235, 1607, and 1640. ^1^H-NMR (methanol-*d*_4_): 6.67 (1H, tt, *J* = 1.6, 6.4 Hz, H-3), 4.49(1H, ddq, *J* = 1.2, 6.4, 13.6 Hz, H-4a), 4.60 (ddt, *J* = 1.2, 6.4, 13.6 Hz, H-4b), 4.15 (1H, q, *J* = 1.2 Hz, H-5), β-d-gluco-pyranosyl moiety: 4.32 (1H, d, *J* = 8.0 Hz, H-1’), 3.20~3.37 (m, glucose proton overlapped by water proton), 3.67 (1H, dd, *J* = 5.6, 12.0 Hz, H-6’a), 3.86 (1H, dd, *J* =1.2, 12.0 Hz, H-6’b). ^13^C-NMR (methanol-*d*_4_): 118.1, 116.8, 144.7, 68.5, 68.5, 63.2, β-d-glucopyranosyl moiety: 104.2, 75, 78.0, 78.0, 71.4, 62.6 ppm. Moreover, the ^1^H-NMR and ^13^C-NMR data of compound **3** are consistent with existing literature [28]. Therefore, on the basis of these data, compound **3** was determined as sarmentosin.

Compound **4**: C_21_H_20_O_11_, yellowish amorphous powder, UV $\lambda_{\max}^{\mathrm{MeOH}}$ nm: 270, 355. ESI-MS: 447 [M − H]^−^, IR ν_max_ cm^−1^: 3359, 2924, 2854, 1655, 1607, 1564, 1512, and 1360. ^1^H-NMR (methanol-*d*_4_): 6.03 (1H, d, *J* = 1.87 Hz, H-6), 6.18 (1H, d, *J* = 1.87 Hz, H-8), 8.03 (2H, d, *J* = 8.96 Hz, H-2’, 6’), 6.84 (2H, d, *J* = 8.96 Hz, H-3’, 5’), β-d-glucopyranosyl moiety: 5.08 (1H, d, *J* = 7.50 Hz 3.18–3.63 (m, glucose proton overlapped by water proton). ^13^C-NMR (methanol-*d*_4_): 159.4, 133.5, 179.9, 161.4, 102.6, 163.3, 95.1, 156.6, 106.2, 121.2, 130.8, 114.6, 160.8, 114.6, 130.8, β-d-glucopyranosyl moiety: 98.7, 73.9, 77.3, 72.4, 78.0, 62.0 ppm. Moreover, the ^1^H-NMR and ^13^C-NMR data of compound **4** are consistent with existing literature [29]. Therefore, on the basis of these data, compound **4** was elucidated to be kaempferol-3-O-β-d-glucopyranoside.

Compound **5**: C_27_H_30_O_16_, faint yellow powder, UV $\lambda_{\max}^{\mathrm{MeOH}}$ nm: 277, 346. ESI-MS: 609 [M − H]^−^, IR ν_max_ cm^−1^: 3385, 1657, 1602, 1589, 1545, 1492, 1415, 1347, 1307, 1283, and 1186. ^1^H-NMR (methanol-*d*_4_): 6.43 (1H, d, *J* = 2.07 Hz, H-6), 6.78 (1H, d, *J* = 2.07 Hz, H-8), 8.05 (2H, d, *J* = 9.0 Hz, H-2’, 6’), 6.88 (2H, d, *J* = 9.0 Hz, H-3’, 5’); β-d-glucopyranosyl moiety: 5.47 (1H, d, *J* = 7.35 Hz, H-1’’), 3.15~3.75 (m); 5.07 (1H, d, *J* = 7.36 Hz, H-1’’’), 3.15~3.75 (m, glucose proton overlapped by water proton). ^13^C-NMR (methanol-*d*_4_): 157, 134, 178.1, 161.4, 99.9, 163.3, 95.1, 156.6, 106.2 121.2, 131.6, 115.8, 160.8, 115.8, 131.6; β-d-glucopyranosyl moiety: 100.25, 74.7, 76.1, 72.4, 75.7, 62.4; 99.9, 74.7, 75.4, 72.2, 75.4, 62.2 ppm. Moreover, the ^1^H-NMR and ^13^C-NMR data of compound **5** are consistent with existing literature [30]. Therefore, on the basis of these data, compound **5** was elucidated to be kaempferol-3,7-di-*O*-β-d-glucopyranoside.

Compound **6**: C_27_H_30_O_15_, yellow amorphous powder UV $\lambda_{\max}^{\mathrm{MeOH}}$ nm: 270, 329. ESI-MS: 593 [M − H]^−^, IR ν_max_ cm^−1^: 3395, 2972, 2850, 1652, 1629, 1582, 1443, 1362, 1286, 1221, and 1181. ^1^H-NMR (methanol-*d*_4_): 6.59 (1H, s, H-3), 7.94 (2H, d, *J* = 8.1 Hz, H-2’, 6’), 6.91 (2H, d, *J* = 8.0 Hz, H-3’, 5’); β-d-glucopyranosyl moiety: 5.01 (1H, d, *J* = 10.8 Hz, H-1’’), 3.5–4.1 (m); 5.04 (1H, d, *J* = 10.8 Hz, H-1’’’), 3.5–4.1 m (m, glucose proton overlapped by water proton). ^13^C-NMR (methanol-*d*_4_): 166.83, 103.89, 184.37, 160.7, 108.2, 161.87, 106.2, 157.65, 105.64, 123.51, 130.3, 117.22, 162.98, 117.22, 130.3; β-d-glucopyranosyl moiety: 75.2, 75.7, 82.8, 72.4, 80.1, 71; 76.4, 75.4, 82.5, 72.2, 79.3, 71 ppm. Moreover, the ^1^H-NMR and ^13^C-NMR data of compound **6** are consistent with existing literature [31]. Therefore, on the basis of these data, compound **6** was identified as vicenin-2.

Compound **7**: C_13_H_18_O_7_, colorless needles, UV $\lambda_{\max}^{\mathrm{MeOH}}$ nm: 223, 273. ESI-MS: 309 [M + Na]^+^, IR ν_max_ cm^−1^: 3500~3200,1615, 1590, and 1575. ^1^H-NMR (methanol-*d*_4_): 7.26 (1H, d, *J* = 8.4 Hz, H-2, 6), 7.06 (1H, d, *J* = 8.4 Hz, H-3, 5), 4.53 (2H, s, H-7); β-d-glucopyranosyl moiety: 4.66 (1H, *J* = 7.8 Hz, H-1’), 3.33–3.49 (m, glucose proton overlapped by water proton), 3.69 (1H, dd, *J* = 5.2, 12.0 Hz, H-6’a), 3.87 (1H, dd, *J* = 2.0, 12.0 Hz, H-6’b). ^13^C-NMR (methanol-*d*_4_): 136.6, 130.9, 118.2, 159.2, 118.2, 130.9, 65.5, 104.2, 74.9, 78.0, 71.3, 77.9, 62.5, 62.5 ppm. Moreover, the ^1^H-NMR and ^13^C-NMR data of compound **7** are consistent with existing literature [32]. Therefore, on the basis of these data, compound **7** was determined as gastrodin.

Compound **8**: White powder, HR-FT-MS [M + H]^+^ *m*/*z*: 487.1812 (calculated at 486.47), UV $\lambda_{\max}^{\mathrm{MeOH}}$ nm: 277, 346, IR ν_max_ cm^−1^: 3362, 2953, 2843, 1660, 1462, 1420, 1123, and 1016. Table 2 lists the ^1^H-NMR and ^13^C-NMR data.

### 3.6. Measurement of Antioxidant Activity

#### 3.6.1. Superoxide Radical Scavenging Activity Assay

Using the method of Lee *et al.*, the superoxide anion scavenging activity of the sample was measured [33]. Through NADH oxidation in a non-enzymatic PMS/NADH system, superoxide anions were generated and assayed through the reduction of NBT. The reagents were prepared in a 100 mM phosphate buffer (pH 7.4). The reaction mixture contained 10 μL of the test sample (1000 ppm), 100 μL of NBT (100 μM), and 100 μL of NADH (468 μM). To this reaction mixture, 10 μL of PMS (60 μM) was added, and the mixture was incubated at room temperature for 15 min. The peak of the UV spectrophotometer changed at 560 nm and recorded the color reaction between the superoxide anion radical and NBT. Trolox was used as a standard for comparative analysis. The reaction mixture without the test sample and without PMS was used as the control and a blank, respectively. Various concentrations of the Trolox solution (20, 80, 121, 181, 222, and 242 μM) were used for plotting a calibration curve. The assay results were expressed as the mean moles of TE per moles of the compounds ± SD, and all analyses were performed in triplicate:

Scavenging activity (%) = [1 − (Abs_sample_)/(Abs_control_)] × 100 (1)

#### 3.6.2. Oxygen Radical Absorbance Capacity Assay

The peroxyl radical scavenging efficacy of the samples was measured using the ORAC assay [34]. A stock solution and dilutions of the test samples were prepared in potassium phosphate solution buffer (75 mmol/L), pH 7.4. Trolox and AAPH were adopted as the standard and peroxyl generator, respectively. Each fluorescein solution (150 μL) (40 nM), 25 μL of AAPH (153 mM), and 25 μL of the sample (1000 ppm) were well mixed. The temperature of the incubator was set at 37 °C for 30 min before measurement, and the fluorescence reading time was recorded every 2 min for 2 h. A fluorescence microplate reader was implemented using an excitation wavelength of 485 nm and an emission wavelength of 528 nm. The areas of the samples under the time and fluorescence intensity were determined by subtracting the area of the blank, and these areas were then compared with those of the standard curve (20, 40, 60, 80, 100, 150, and 220 μM). The assay results were expressed as the mean moles of TE per moles of the compounds ± SD, and all the analyses were performed in triplicate. 

#### 3.6.3. Assay on Chelation of Ferrous Ions

The chelation of the ferrous ions of the sample was estimated using the method of Lim *et al.* [35]. The tested sample solutions (10 μL) were added to a solution of 2.0 mM ferrous chloride (10 μL) and methanol (370 μL). The reaction was initiated by adding 5 mM ferrozine (20 μL), and this mixture was then vigorously shaken and maintained at room temperature for 10 min. The absorbance of the resulting solution was recorded at 562 nm. Various concentrations of the Trolox solution (12.5, 50, 100, 125, 175, and 220 μM) were used for plotting a calibration curve. The assay results were expressed as the mean moles of TE per moles of the compounds ± SD, and all the analyses were performed in triplicate.

Ferrous ion-chelating ability (%) = [1 − (Abs_sample_)/(Abs_control_)] × 100 (2)

#### 3.6.4. Assay on Inhibition of Nitric Oxide Radical 

NO generated from aqueous SNP at physiological pH interacted with oxygen to produce nitrite ions, which were measured according to the Griess reaction [36]. NO scavengers compete with oxygen, possibly reducing the production of NO [37]. Aqueous SNP (5 mM, approximately 200 μL) and 0.2 M potassium phosphate buffer (pH 7.4, 200 μL) were added to the test sample (1000 ppm, 200 μL). After incubation for 150 min at 25 °C, sulfanilamide (15 [*w*/*v*], 200 μL) was added to the incubated solution (600 μL) and allowed to stand for 10 min. Subsequently, NED (0.1% [*w*/*v*], 200 μL) was added, and the mixture was incubated for 20 min at 25 °C. The absorbance of chromophores was recorded at 546 nm against a blank sample. Various concentrations of the Trolox solution (12.5, 37.5, 50.0, 62.5, and 75.0 μM) were used for preparing a calibration curve. The assay results were expressed as the mean moles of TE per moles of the compounds ± SD, and all the analyses were performed in triplicate.

Nitric oxide radical scavenging (%) = [1 – (Abs_sample_)/(Abs_control_)] × 100 (3)

### 3.7. Comparison of Chromatograms after Isopropanol Salting-Out Pretreatment and n-Butanol Partition Extractions Using HPLC Separation

An appropriate volume of methanol was used to individually dissolve compounds **5** and **6** to prepare 1000 μg/mL stock solutions. The mixture of each stock solution (100 μL) was used to prepare a standard working solution that contained 500 μg/mL of each compound. Reversed-phase chromatography was performed on compounds **5** and **6** (Merck C18 column [LiChriCART 5 μm 250-4 RPC18e] and on a Phenomenex C18 guard column [AJ0-4287 4.0 × 3.0]). The crude extracts were separated under the following experimental conditions: eluent flow rate of 0.4 mL/min; injection volume of 20 μL; detection wavelength of 210 nm; ambient temperature; and an eluent of water (A) and ACN (B) mixtures. Using a linear gradient, the elution program was optimized as follows: 0 min, 0% B; 120 min, 100% B; 125 min, 100% B; and 135 min, 0% B. The retention times (t_R_, min) of compounds **5** and **6** were 19.0 and 20.0 min, respectively. An appropriate volume of methanol was used to individually dissolve the crude extracts of ISP and BP extraction to obtain 1000 μg/mL work solutions. Chromatograms of these individual work solutions were obtained under identical experimental conditions for HPLC.

### 3.8. Statistical Analysis

Values are represented as the mean ± SD of three parallel experiments and were analyzed through a *t-*test and analysis of variance.

## 4. Conclusions

This investigation demonstrated that the antioxidant capability of compounds obtained through ISP extraction was 1.38–3.65 times higher than that of compounds obtained through BP extraction in antioxidant assays. Eight compounds, namely three flavonoid glycosides, three cyanogenic glycosides, and two phenolic compounds, were extracted and isolated from *S. formosanum* N.E.Br. through the ISP method. Compound **8** is a new compound. Except for the inhibition of NO activity, the antioxidant capability of compound **6** was 1.03–1.23 times higher than that of the standard compound (Trolox). According to the HPLC chromatograms, ISP extraction had the highest efficiency to extract compounds **5** and **6**. Furthermore, hydrophilic ISP extraction technology is superior to BP extraction technology regarding antioxidant capability and cost and reduces the risk to human health and the environment. Therefore, ISP extraction technology must replace BP extraction technology for extracting and isolating higher polar compounds of natural products. This study devised an effective method of extracting and identifying active compounds for *S. formosanum* N.E.Br. that can be used as nutraceuticals and pharmaceuticals.

## Figures and Tables

**Table 1:** Results of the different free radical scavenging capacities obtained using the isopropanol salting-out and *n*-butanol partition extraction methods for *Sedum formosanum* N.E.Br. Values represent means ± SD (*n* = 3).

	Extraction Method	Isopropanod Salting-Out Extraction Method		Butanol Partition Extraction Method	
Antioxidant Assay		Average Cleaning Factor (%)	Equivalent to Trolox (mmol/g) ^a^	Average Cleaning Factor (%)	Equivalent to Trolox (mmol/g) ^a^
^b^ FRAP		31.85	0.071 ± 0.011	21.60	0.052 ± 0.005
^c^ SOD		77.96	0.210 ± 0.005	20.41	0.057 ± 0.007
^d^ NO* scavenging		57.48	0.118 ± 0.006	25.27	0.054 ± 0.007
^e^ ORAC		70.56	0.198 ± 0.005	57.83	0.118 ± 0.005

^a^ (mmol/g): are expressed micromoles of Trolox equivalent to per gram of the test dried materials weight. ^b^ FRAP: Ferrous ion chelating activity. ^c^ SOD: Superoxide anion radical scavenging activity. ^d^ NO* scavenging: Nitric Oxide radical scavenging activity. ^e^ ORAC: Oxygen Radical Absorption Capacity

**Table 2:** ^1^H-NMR (400 MHz) spectra and ^13^C-NMR (100 MHz) spectra of compound **8**.

Position	Compound 8 (in CD_3_OD)	
	^1^H (δ) (ppm)	^13^C (δ) (ppm)
1		158.4
2	7.06 (1H, d, *J* = 8.8 Hz)	116.4
3	7.67 (1H, d, *J* = 8.8 Hz)	132.7
4		130.9
5	7.67 (1H, d, *J* = 8.8 Hz)	132.7
6	7.06 (1H, d, *J* = 8.8 Hz)	116.4
7	6.93 (1H, d, *J* = 12.8 Hz)	144.2
8	5.88 (1H, d, *J* = 12.8 Hz)	117.8
9		168.3
10	3.66 (3H, s)	51.8
Rha		
1’	5.51(1H, d, *J* = 1.8 Hz)	99.4
2’	4.30 (1H, m)	71.3
3’	3.97 (1H, m)	82.7
4’	3.95 ^a^ (1H, m)	72.6
5’	3.67 ^a^ (1H, m)	70.4
6’	1.23 (3H, d, *J* = 6.0 Hz)	18.1
Glc		
1”	4.61 (1H, d, *J* = 7.5 Hz)	105.9
2”	3.35 ^a^ (1H, m)	72.3
3”	3.39 ^a^ (1H, m)	77.7
4”	3.66 ^a^ (1H, m)	72.6
5”	3.37 ^a^ (1H, m)	77.8
6’’a	3.74 ^a^ (1H, dd, *J* = 11.9; 4.7 Hz, 1H)	62.0
6”b	3.85 ^a^ (1H, dd, *J* = 11.9; 2.31 Hz, 1H)	

^a^: Overlapping with other signals.

**Table 3:** Antioxidant activities of pure compounds isolated from the isopropanol layer of *Sedum formosanum* N.E.Br. Values represent means ± SD (*n* = 3).

Compounds	FRAP (mol of TE/mol) ^a^	SOD (mol of TE/ mol) ^a^	NO* Scavenging (mol of TE/ mol) ^a^	ORAC (mol of TE/mol) ^a^
**1**	0.17 ± 0.01	0.25 ± 0.01	0.09 ± 0.04	nt ^c^
**2**	0.33 ± 0.02	0.53 ± 0.01	nd ^b^	nt ^c^
**3**	0.29 ± 0.01	0.19 ± 0.01	nd ^b^	nt ^c^
**4**	0.91 ± 0.04	0.81 ± 0.04	0.59 ± 0.02	0.82 ± 0.04
**5**	1.14 ± 0.01	0.95 ± 0.03	1.01 ± 0.05	1.03 ± 0.03
**6**	1.23 ± 0.01	1.03 ± 0.04	0.83 ± 0.02	1.06 ± 0.06
**7**	0.82 ± 0.02	1.21 ± 0.07	0.32 ± 0.01	nt ^c^
**8**	0.60 ± 0.02	0.51 ± 0.07	0.48 ± 0.05	0.64 ± 0.02
Trolox (reference) 1.00 ± 0.01				

mol of TE/mol ^a^: are expressed as mole of Trolox equivalent per pure compound mole. Values represent means ± SD (*n* = 3). nd ^b^: Not detected or lower than the calibration curve range. nt ^c^: Not test in the antioxidant assay.
